# Atrial fibrillation—a complex polygenetic disease

**DOI:** 10.1038/s41431-020-00784-8

**Published:** 2020-12-05

**Authors:** Julie H. Andersen, Laura Andreasen, Morten S. Olesen

**Affiliations:** 1 Laboratory for Molecular Cardiology, Centre for Cardiac, Vascular, Pulmonary and Infectious Diseases, Rigshospitalet, University Hospital of Copenhagen, Copenhagen, Denmark; 2 Department of Biomedical Sciences, University of Copenhagen, Copenhagen, Denmark

**Keywords:** Atrial fibrillation, Genetics

## Abstract

Atrial fibrillation (AF) is the most common type of arrhythmia. Epidemiological studies have documented a substantial genetic component. More than 160 genes have been associated with AF during the last decades. Some of these were discovered by classical linkage studies while the majority relies on functional studies or genome-wide association studies. In this review, we will evaluate the genetic basis of AF and the role of both common and rare genetic variants in AF. Rare variants in multiple ion-channel genes as well as gap junction and transcription factor genes have been associated with AF. More recently, a growing body of evidence has implicated structural genes with AF. An increased burden of atrial fibrosis in AF patients compared with non-AF patients has also been reported. These findings challenge our traditional understanding of AF being an electrical disease. We will focus on several quantitative landmark papers, which are transforming our understanding of AF by implicating atrial cardiomyopathies in the pathogenesis. This new AF research field may enable better diagnostics and treatment in the future.

## Introduction

Atrial fibrillation (AF) is the most common cardiac arrhythmia. It affects more than 33 million people worldwide and is associated with a significant increase in morbidity and mortality. The life time risk has been estimated to 25% [1]. AF causes irregular and often abnormally fast heart rate. Patients with AF have an increased risk of heart failure, stroke, dementia, and death [2]. Risk factors for AF include increasing age, heart disease, high blood pressure, and alcohol overuse [1]. In other instances, no risk factors can be identified, which suggests an underlying genetic predisposition to AF. The pathogenesis of AF remains poorly understood, which, to some degree, compromises the development of effective treatments.

The underlying AF mechanisms have been subject to intense research. AF predominantly arise secondary to hypertension, ischemic and/or structural heart disease [2]. These cardiovascular diseases can influence the electrical and structural remodeling of the atria which is likely to play a central role in the pathogenesis of AF. It has furthermore been recognized that atrial myopathy and fibrosis seem to play a driving role in the development of AF [3, 4].

Arnar et al. performed a population-based cohort study of more than 5000 Icelandic AF patients in 2006. The study demonstrated substantial familial aggregation of AF and a strong likelihood of heritability among AF patients. At the time, this led to the likely assumption that there may be undiscovered genetic variants underlying the risk of common AF in Iceland [5]. In 2009, a large study was completed in 1137 Danish twins, investigating the heredity of AF. The heritability of AF was found to be 62% based on biometric models [6]. In 2012, an epidemiological study investigated whether an individual’s risk of developing AF without having risk factors for AF (previously termed lone AF) before 60 years is associated with familial lone AF. This study found an increased risk of lone AF when having family members with lone AF, with the strongest risk associated with multiple affected relatives and relatives with a young age at AF onset [7]. The epidemiological results support that genetic factors seem to contribute to the risk of AF.

Through linkage and family studies, rare AF-associated variants were initially identified to locate to genes encoding ion channels, such as the potassium and sodium channels, contributing to cardiac depolarization or repolarization. Rare variants in ion and non-ion-channel genes were later identified through candidate gene studies, which recognized several rare variants based on prior knowledge of the specific genes.

Genome-wide association studies (GWAS) on AF have shown that more than a hundred single nucleotide polymorphisms (SNPs) also contribute to the risk of AF [8]. The remaining heritability of AF may be explained by promoter variants, epigenetics, structural variants and undiscovered genetic mechanisms [9].

In this review, we will focus on the genetic basis of AF, the role of both common and rare genetic variants and the link to cardiomyopathy and atrial fibrosis.

## Materials and methods

We conducted an extensive review of the literature through the online library PubMed. We performed a systematic literature search with the query ((“Atrial fibrillation” [MeSH]) OR (atrial fibrillation)) AND ((“Genetics” [MeSH]) OR (genetic*)) AND ((“Mutation” [MeSH]) OR (mutation*) OR (“Polymorphism, single nucleotide” [MeSH]) OR (polymorphism, single nucleotide) OR (monogenic*) OR (GWAS)). Articles concerning the genetic basis of AF were included and small studies concerning common variants in genes not associated with AF were excluded from this review. Common and rare genetic variants were investigated in various publicly available gene variant databases; gnomAD, TransVar, Ensembl, UCSC Genome Browser, Mutalyzer and NCBI databases [10–15]. Details on rare genetic variants including annotation, original study type, transcript and country of origin were found in the original studies if present or investigated in the beforementioned databases (see Supplementary Table 2).

## Results and discussion

### The role of common genetic variants

AF has been subject to intense research and investigators have identified many genetic loci associated with AF [16]. A list of common variants associated with AF is shown in Supplementary Table 1.

The most significantly associated AF SNP in all of these studies is located in a noncoding region of chromosome 4q25, ~150,000 base pairs upstream of the gene encoding the paired-like homeodomain transcription factor pitx2 [8]. *PITX2* encodes three different isoforms; pitx2a, pitx2b and pitx2c, where pitx2c is the most prominent isoform in the developing heart [17]. Previous work has indicated that *PITX2* expression in humans has been significantly reduced in AF patients, offering a connection between *PITX2* loss-of-function and AF [17]. In a recent paper by Collins et al. *PITX2* loss-of-function was investigated in CRISPR-Cas9 modified zebrafish. These animals were found to have a compromised sarcomere, an increased amount of fibrosis and a more than fourfold upregulation of the expression of the pacemaker gene *HCN4*. An increased expression of *HCN4* may be part of the explanation for increased ectopic activity seen in AF patients [18].

Another highly significant SNP identified through GWAS is rs2106261 (NC_000016.9 (NM_001164766.2:g.73051620C>T)) located on chromosome 16q22 intronic to the transcription factor gene *ZFHX3* [19]. The function of ZFHX3 in cardiac tissue is unknown, but it is expressed in mouse hearts and has been associated with myogenic and neuronal differentiation [20].

The SNP rs13376333 (NC_000001.10 (NM_ 002249.6:g.154814353C>T)) is strongly associated with AF, and has been located to an intron of *KCNN3* on chromosome 1q21. This gene encodes a calcium activated potassium channel (the SK3 channel) and is thought to be involved in atrial repolarization [21]. Interestingly, there is currently an ongoing clinical phase II study targeted towards inhibition of SK3 channels. These ion channels are present in the heart, where they play a role in regulating the cardiac rhythm. Blocking these channels leads to an antiarrhythmic effect by prolonging the action potential in the atria selectively. This is a promising approach for a new effective treatment of AF, that might prevent harmful pharmaceutical side effects in the ventricles [22]. Furthermore, the gene *KCNN2* encodes the SK2 channels which have been shown to co-assemble with the SK3 channels and the gene has been associated with AF in a recent GWAS [16].

Hyperpolarization-activated cyclic nucleotide-gated (HCN) pacemaker channels are expressed in the heart and the gene *HCN4* is the predominant gene encoding the cardiac pacemaker channels [23]. This gene has been associated with AF in GWAS [24].

Another locus associated with AF has been found on chromosome 10q22 (rs10824026; NC_000010.10 (NM_001114133.3:g.75421208G>A)) located 5 kb upstream of *SYNPO2L* and 20 kb upstream of *MYOZ1*. The structural proteins encoded by these genes are both expressed in skeletal and cardiac muscle, however it is unknown which of these genes are driving the association [24].

In 2018, Thorolfdottir et al. reported an association between AF and two common variants in the gene *RPL3L* on chromosome 16 and one variant in the *MYZAP* gene on chromosome 15. The *RPL3L* gene encodes a ribosomal protein, primarily expressed in skeletal and heart muscle. *MYZAP* encodes myozap, which is a myocardial zonula adherence protein, mainly expressed in the human heart. The mouse homolog has been located to the intercalated discs [25]. Seeger et al. were the first to identify myozap and because of both the subcellular localization within the cardiomyocytes and the observation of a severe cardiac phenotype in mutated zebrafish, myozap is thought to be implicated in a subtype of atrial cardiomyopathy [26].

In 2017, Christophersen et al. identified 12 novel AF loci through GWAS, implicating genes involved in cardiac and structural remodeling [27]. Later, an AF GWAS was completed in 2018 by Nielsen et al. identifying one novel risk locus and confirming 13 out of 16 already known AF loci [28]. The most statistically significant novel association was observed at the 2q31 locus harboring seven highly correlated missense variations. These missense variants fell within the I-, A- and M-bands of *TTN*, a strong biological AF candidate gene because of its role in the structural integrity and muscle elasticity of the heart [28]. We will discuss the role of *TTN* in more detail in a later section.

Same year, a large AF GWAS meta-analysis was conducted which reported a more than threefold increase in the number of loci associated with AF [29]. The analysis was conducted in over half a million individuals, including 65,446 with AF, from more than 50 studies. Two identified loci were located close to genes that are targets for current antiarrhythmic medications. *SCN5A*, which will be mentioned in the next section, encodes the Nav1.5 channel which is the target for sodium channel blockers such as flecainide. Similarly, *KCNH2* encodes the Kv11.1 channel which is the target for potassium-channel-inhibiting medications such as amiodarone. The GWAS furthermore proved transcriptional regulation to be a key feature in AF by associating loci close to genes encoding transcription factors, e.g., *TBX3*, *TBX5* and *NKX2-5*, with AF. These genes are involved in the development of the cardiac conduction system [29].

Later in 2018, Nielsen et al. conducted an even larger meta-analysis and identified AF risk variants located close to genes which have been associated with serious heart defects in humans, e.g., *GATA4*, *MYH6*, *NKX2-5*, *PITX2*, or near genes important in striated muscle function e.g., *MYH7* [16]. One-hundred-and-eleven genomic regions were identified with at least one variant associated with AF. Furthermore, the authors found an association between younger age of AF onset and a high genetic burden of AF risk variants [16].

Interestingly, the GWAS approach has implicated many genes already suspected to be involved in AF through candidate gene studies (e.g., *GJA5*, *KCNH2*, *SCN5A*, *KCNJ2*, *KCND3*, *MYH7*, and *NKX2-5*). AF GWAS have furthermore identified AF-associated genes which have been implicated in a variety of inherited arrhythmias, other conduction diseases and cardiomyopathies. This highlights the pleiotropy of these genes as well as the polygenetic nature of AF.

### The role of rare genetic variants

**Fig. 1:**
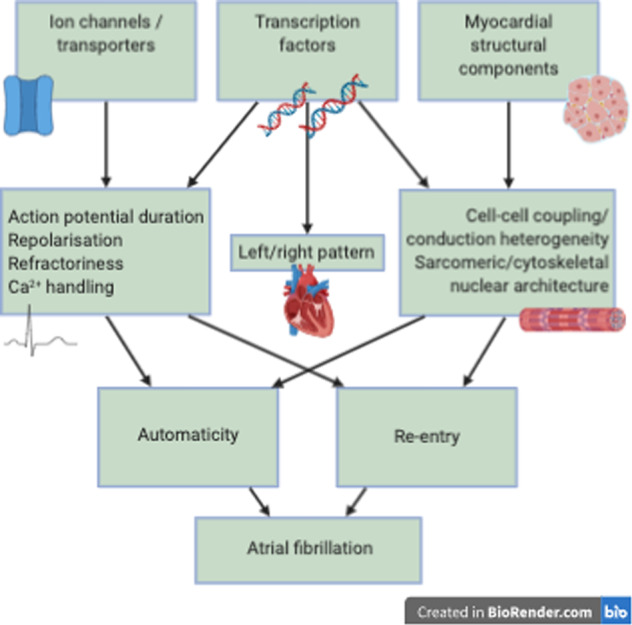
AF mechanisms. Genetic variants in genes encoding different ion-channels, transcription factors and myocardial structural components predispose to atrial fibrillation (AF) through different pathways that lead to increased automaticity and reentry activity.

Rare variants in genes encoding ion channels, signaling molecules, accessory subunits and gap junctions have been associated with AF. These variants can lead to AF through different pathways, which is illustrated in Fig. 1. Rare AF-associated variants in different genes are listed in Supplementary Table 2 and will be presented in the following.

#### Potassium-channel variants

The gene *KCNQ*1 encodes the pore-forming α-subunit of the cardiac potassium-channel I_Ks,_ which is involved in cardiac cell repolarization. The first association between rare variants in *KCNQ1* and familial AF was discovered in 2003 and functional analysis of the c.418A>G (p.(Ser140Gly)) variant showed a gain-of-function effect [8]. As opposed to I_Ks_, which has six transmembrane domains, the β-subunits of I_Ks_ are single-transmembrane units and are encoded by the *KCNE* genes, *KCNE1-KCNE5*. Isolated and familial cases with AF have been shown to carry rare variants in the β-subunits of the I_Ks_ channel [8].

The gene *KCNH2* encodes the α-subunit of the I_Kr_ channel and a rare variant in this gene has been identified in a family with AF and short QT, proposing an overlap in phenotypes [8]. Several other variants have been discovered, which are shown in Supplementary Table 2.

The inward rectifier channel Kir2.1 mediates the I_K1_ current involved in repolarisation and is encoded by the gene *KCNJ2* (Supplementary Material reference 21). A missense variant was found in *KCNJ2* in a Chinese AF kindred. Functional analysis demonstrated a gain-of-function variant, and the authors hypothesized that the gene may play a role in initiating and/or maintaining AF [8]. Additional variants are shown in Supplementary Table 2.

A missense variant was found in a cohort of AF patients in the gene *KCNJ8*, which encodes the cardiac K_ATP_ channel Kir6.1 [8]. The authors suggested that the variant was associated with increased susceptibility to AF and the variant has shown to give rise to a gain-of-function effect of the cardiac channel [8].

A particularly interesting case is *KCNA5*. *KCNA5* encodes an atria specific K_v_1.5 channel involved in cardiac repolarisation and several loss-of-function variants have been identified and associated with AF [8]. Christophersen et al. identified six novel rare variants in *KCNA5* in seven patients with early-onset lone AF. The authors found rare variants in this gene to lead to a gain-of-function effect and hence support the hypothesis that both gain- and loss-of function of the I_Kur_ current enhance AF susceptibility [30].

Olson et al. [31] identified a missense variant in *ABCC9*, which encodes a K_ATP_ channel subunit. The missense variant was found in a female with early-onset AF (Supplementary Material reference 1).

#### Sodium channel variants

*SCN5A* encodes the cardiac sodium channel Na_v_1.5 and several *SCN5A* variants have been reported to associate with AF [32]. In 2012, Olesen et al. identified ten rare variants in *SCN5A* in a cohort of 192 patients with early-onset AF [33]. Many of these lone AF patients carried an *SCN5A* variant previously associated with Long QT syndrome (LQTS). Functional investigations of the *SCN5A* variants revealed both compromised transient peak current and increased sustained current, indicating both gain- and loss-of-function variants in early-onset AF [33]. Several *SCN5A* variants have subsequently been identified in AF patients and are listed in Supplementary Table 2.

Variants in the four sodium channel ß-subunits, ß1-4, encoded by *SCN1-4B*, have been identified in AF patients. Loss-of-function variants were identified in *SCN1-2B* in a cohort of AF patients [34]. Furthermore, another study found three loss-of-function variants in *SCN3B* in a lone AF cohort, indicating that this gene contribute to the mechanisms behind lone AF [35].

*SCN10A* encodes the voltage-gated Na_v_1.8 channel and has been associated with the electrocardiographic (ECG) PR-interval and AF [36]. Jabbari et al. found ten rare missense *SCN10A* variants in 225 lone AF patients and showed that the common variant c.3218T>C (p.(Val1073Ala)) (rs6795970; NC_000003.11 (NM_001293307.2:g.38766675A>G)) increased the risk of AF [36]. Furthermore, functional characterization of the rare variants revealed reduced activity in Na_v_1.8, while the common c.3218T>C (p.(Val1073Ala)) variant had a gain-of-function phenotype, which seems to increase the risk of AF. These data on rare variants and the common c.3218T>C (p.(Val1073Ala)) variant suggest that both gain- and loss-of-function of the Na_v_1.8 current could be involved in the development of AF [36], but further research is needed to confirm the involvement of rare variants in *SCN10A*. The variants are summarized in Supplementary Table 2.

#### Non-ion channel variants

Zhang et al. discovered a homozygous variant, c.1172G>A (p.(Arg391His)) in *NUP155*, that co-segregated in an AF family. Furthermore, heterozygous *NUP155*(+/−) knockout mice were shown to have AF-like ECG recordings [37].

The *NUP155* gene is localized to the chromosome 5p13 and encodes a nucleoporin, a main component of the nuclear pore complexes involved in cytoplasmic transport [37]. Oberti et al. [38] identified a large AF family with an autosomal recessive inheritance pattern and found a co-segregating variant in a locus at chromosome 5p13 (Supplementary Material reference 115). This finding provides evidence that *NUP155* might be a disease-causing AF gene.

Hodgson-Zingman et al. studied a family with AF segregating as an autosomal dominant trait and a co-segregating frameshift variant in *NPPA* [39]. *NPPA* encodes the atrial natriuretic peptide (ANP). ANP is a circulating hormone secreted from the cardiac atria involved in the regulation of blood pressure [39].

GATA-4 and GATA-6 are cardiac transcription factors involved in myocardial development. In 2010, Posch et al. found variants in the *GATA4* gene in a patient with familial lone AF and a second variant in a patient with sporadic AF [40]. Other studies have identified additional *GATA4* variants that co-segregate with AF and Yang et al. identified two heterozygous loss-of-function *GATA6* variants. These rare variants co-segregated with AF as an autosomal dominant trait [41].

The AF-associated gene, *GREM2*, encodes the bone morphogenic protein antagonist gremlin-2. Functional studies in zebrafish have revealed that *GREM2* is required for cardiac laterality and atrial differentiation during embryonic development [42]. Furthermore, a live heart imaging study in a zebrafish overexpressing the *GREM2* variant, c.226C>G (p.(Gln76Glu)), showed an abnormal contraction velocity specifically in atrial cardiomyocytes suggesting that the gene could play a role in AF [42].

#### Gap-junction variants

Connexin43 and connexin40 are encoded by *GJA1* and *GJA5*, respectively, and are gap-junction proteins in the atrial myocardium. A study done by Thibodeau et al. identified a novel loss-of-function somatic variant in *GJA1* associated with lone AF [43]. In contrast to this finding, Gregers et al. investigated the prevalence of somatic variants in 44 AF patients undergoing mitral valve surgery [44]. This much larger study did not identify any somatic variants, indicating that somatic variants do not play a major role in the pathogenesis of AF. Interestingly, a high proportion of the patients in the cohort had rare germline variants indicating that these non-lone AF patients, may also be predisposed to AF by rare germline variants [44]. Several *GJA1* and *GJA5* variants are shown in Supplementary Table 2.

#### Variants affecting cardiac structure

Other rare variants in the genes *MYH7*, *MYBPC3*, *MYL4*, and *TTN* have been associated with AF and cardiomyopathy and will be explained in a later section [45–47].

As described above, rare variants in genes encoding different ion channels, transcription factors as well as structural components of the myocardium have been associated with AF. Figure 1 illustrates how rare variants can lead to atrial pathology e.g., altered sarcomeric architecture, which in turn may lead to arrhythmias through reentry or automaticity.

### Genetic overlap with other diseases

AF has been associated with other phenotypes in patients with inherited arrhythmia syndromes, such as Brugada syndrome (BrS) and LQTS, but also with familial cardiomyopathies such as hypertrophic cardiomyopathy (HCM) and dilated cardiomyopathy (DCM).

LQTS is a cardiac repolarization abnormality and has been associated with variants in genes such as *KCNQ1*, *KCNE1-3*, *KCNH2*, *KCNJ2*, and *SCN5A* [48]. Patients with genetically proven LQTS have a higher risk of early-onset AF than the rest of the population [49]. Nielsen et al. have previously found that both shortened and prolonged QTc interval durations are risk factors for AF in the general population. The association was strongest in lone AF patients, suggesting a link between an extreme QTc interval and AF [50]. BrS is an inherited syndrome associated with a high incidence of sudden cardiac arrest and has been linked to rare variants in different genes e.g., *KCNE3*, *KCNE5*, *KCND3*, *KCNH2*, *SCN5A*, *SCN1Bb*, and *SCN3B* [51].

A recent GWAS on coarctation of the aorta among Icelanders (120 cases and 355,166 controls) identified a rare missense variant in *MYH6* which also associated with Sick Sinus Syndrome (SSS) and AF [52]. *MYH6* encodes an alpha myosin heavy chain subunit (α-MyHC). Myosin is an essential component of the cardiac muscle and α-MyHC is a fast motor protein of the thick filaments of the contractile apparatus in healthy adult atrial muscle [53]. *MYH7*, on the other hand, encodes a slower MyHC motor protein, β [54]. The β-MyHC is upregulated in heart failure and other cardiac disorders, whereas α-MyHC is downregulated, giving rise to the hypothesis that MyHC isoforms play a role in the determination of cardiac contractility [55]. New research has established a link between an increased *MYH7* expression in atrial tissue from patients with chronic AF. Chronic AF myofibrils show biochemical and biophysical differences from healthy myofibrils, potentially as a result of increased *MYH7* expression [56].

Another Icelandic study identified a variant in *PLEC* to associate with both a 55% increased risk of AF and a 64% increased risk of SSS. This gene encodes structural components of the cardiomyocyte [57].

The human genome is contained within the cell nucleus and alterations in the nuclear envelope protein lamin A/C, encoded by the gene *LMNA*, cause a number of diseases such as cardiomyopathy and muscular dystrophy [58]. A heterozygous missense rare variant was identified in a family with AF, as well as supraventricular tachycardia, ventricular tachycardia and sudden cardiac death, in 2010 by Beckmann et al. [59]. See Supplementary Table 2 for a list of *LMNA* variants.

In 2018, Bundgaard et al. identified five families with an autosomal dominant cardiac syndrome characterized by uniform ECG changes with persistent non-ischemic ST-segment depressions, AF and ventricular arrhythmias [60]. The ST-segment depression remained stable over time, in contrast to other genetic disorders (BrS and LQTS), that are characterized by dynamic pathognomic ECG changes. Genetic evaluation was performed, however no coding variants were identified to associate with the syndrome [60].

Hyperthyroidism is well known to contribute to cardiovascular morbidity, particularly AF [61]. A mendelian randomization study by Salem et al. suggests a genetically determined variation in thyroid function within a physiologically normal range as a risk factor for AF [61]. In 2019, Ellervik et al. found an association between genetically increased FT3:FT4 ratio and hyperthyroidism and AF [62]. Thyroid hormone replacement for hypothyroidism may increase the AF risk, whereas antithyroid medications to treat hyperthyroidism may reduce the risk of AF. This complicates the treatment of patients with subclinical thyroid disease, and the risk of AF should probably be considered in the clinical decision to treat these patients [61].

**Fig. 2:**
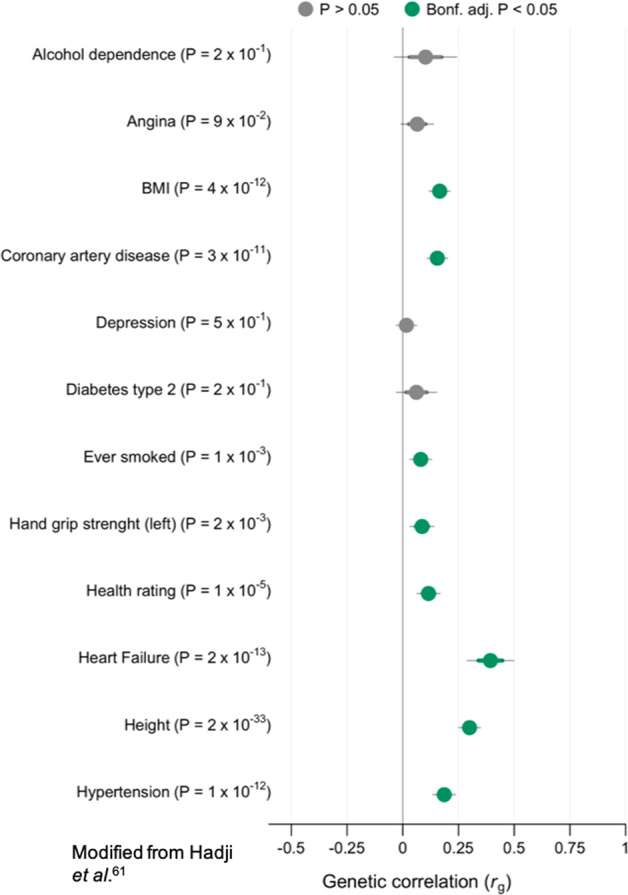
Genetic correlation with AF. LD score regression revealing genetic correlation between AF (65,446 cases and >500,000 controls) and other phenotypes. Phenotypes with negative log10(P) are displayed on the *y*-axis. *X*-axis show genetic correlation (rg). Dots are estimated values with thick lines indicating mean standard error (SE) and thin lines 1.96 SE. Significant association after Bonferroni corrections is denoted with green color. Nominal significance is denoted with violet color and non-significant correlations with grey. SEs and *p* values were derived from using block jackknife resampling. Modified from Hadji-Turdeghal et al. [63].

Genetic correlation between AF and other traits is illustrated in Fig. 2 (modified from Hadji-Turdeghal et al. [63]). The most significant genetic correlation with AF is heart failure (*p* = 2 × 10^−13^). Height was highly correlated with AF (*p* = 2 × 10^−33^) and so was hypertension, a risk factor for AF (*p* = 1 × 10^–12^). Interestingly, other well-established risk factors for AF such as diabetes type 2 and alcohol dependence, had a non-significant correlation with AF (*p* = 2 × 10^−1^).

#### Cardiomyopathy

There is an increasing interest in cardiomyopathy and how it is linked to AF. The relationship between genetic variation in HCM and AF has been poorly described, although AF is common in cardiomyopathy patients [64]. Factors such as age and left atrium (LA) size are predictors for AF in HCM patients, where larger LA size may increase the left ventricle (LV) diastolic pressure resulting in diastolic dysfunction [64]. Cardiomyopathies, in particular arrhythmogenic right ventricular cardiomyopathy (ARVC), have been associated with variants in the intercalated disc genes and ARVC patients have an increased risk of AF and ventricular arrhythmias. This is a leading cause of sudden death in young people and athletes [65]. Many rare variants are linked to HCM, but variations in the *MYH7* and *MYBPC3* genes are the most common [45]. It is still unclear why HCM patients with *MYH7* rare variants have a higher risk of AF, but variations in this gene may cause extensive myocardial disease leading to reduced cardiac performance, which may be associated with higher occurrence of AF [64].

Recently, Vad et al. identified six individuals with rare loss-of-function variants in three different DCM genes (*DMD*, *PDLIM3*, and *FKTN*), of which two variants were novel. These data suggest that rare loss-of-function variants in cytoskeletal genes previously associated with DCM may have a role in early-onset AF, perhaps through the development of an atrial cardiomyopathy [66].

Several findings suggest that AF could be caused by atrial cardiomyopathy [67]. Peng et al. showed an association between the *MYL4* gene and atrial cardiomyopathy. The *MYL4* gene is responsible for the electrical, contractile and structural integrity of the atrium. A loss-of-function variant in the *MYL4* gene was found to cause early atrial fibrosis leading to atrial cardiomyopathy and atrial arrhythmia, but also atrial contractile failure and atrial enlargement [46]. Furthermore, two other studies have independently identified an association between early-onset AF and a rare variant in *MYL4* [3, 68]. Gudbjartsson et al. [3] identified a frameshift variant in *MYL4* associated with a recessive form of AF. The authors found a high risk of stroke in these patients, potentially related to underlying atrial cardiomyopathy. When assessed by the CHAD2DS2-VASc score, the patients were unexpectedly scored to having a low stroke risk, suggesting an alternative stroke mechanism. The results raise the possibility of a genotype-based risk stratification of atrial thrombus formation.

*TTN* is thought to be an important AF gene, as mentioned previously. The gene encodes a giant sarcomere protein (titin) expressed in all chambers of the human heart and titin-truncating variants (TTNtv) have been shown to predispose directly for AF [47]. TTNtv are known to occur in about 15% of DCM cases and independently predict early arrhythmias in DCM patients [69]. In 2018, Choi et al. found loss-of-function variants in *TTN* to be associated with early-onset AF. The study supports the role of malfunctioning sarcomeric proteins in the pathogenesis of AF [70]. The occurrence of *TTN* loss-of-function variants in AF and DCM patients suggests that impaired sarcomere function may be an overlapping pathophysiological mechanism.

Ahlberg et al. found a significant enrichment of rare TTNtv in families diagnosed with AF (*n* = 399; odds ratio = 36.8; *p* = 4.13 × 10^−6^) [47]. Using a zebrafish model carrying a rare variant in ttn, the homologs *TTN* gene in zebrafish, the authors showed a distinct sarcomere defect in the mutants. Further analysis of the heart revealed an increased amount of fibrosis and a compromised sarcomere structure in the mutant larvae and adult fish, suggesting a predisposition for arrhythmia and conduction disease. The discovery of atrial fibrosis in young zebrafish, indicates that TTNtv predispose to the development of fibrosis in the atria from an early age. The zebrafish findings and the early-onset of AF in the replication cohort both propose a possible link between structural disease and electrical phenotype [47].

**Fig. 3:**
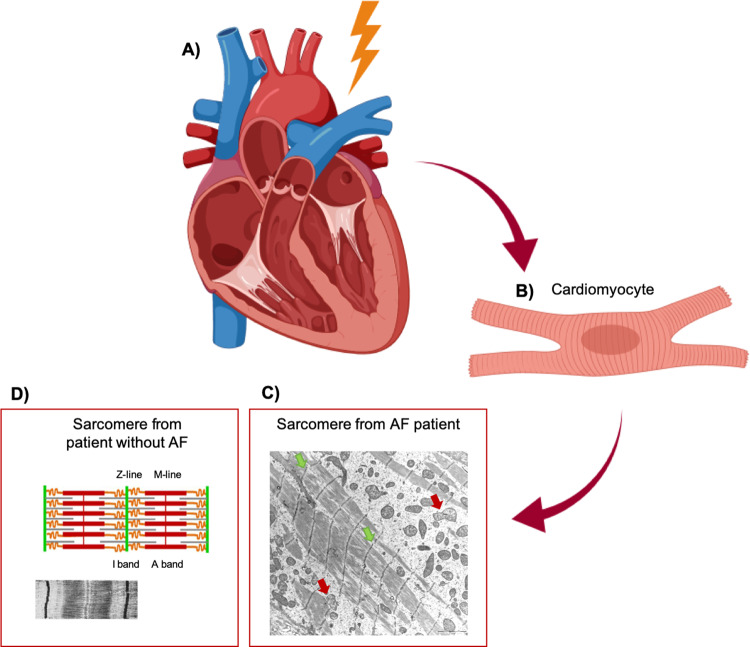
AF and atrial cardiomyopathy. Structural rearrangement in the heart seems to play a key role in atrial cardiomyopathy and in AF, here illustrated with a transmitted electron microscopy (TEM) image of the cardiomyocyte of a patient with AF and atrial cardiomyopathy. **A** Heart and **B** cardiomyocyte from AF patient with atrial cardiomyopathy, and **C** TEM imaging of sarcomere from AF patient with atrial cardiomyopathy affected by fibrotic changes (light areas). The sarcomeres look disrupted with poorly defined M-lines and I-bands, and fuzzy Z-lines (green arrows) and the mitochondria show an increased amount of cristae and ballooning (red arrows). **D** For comparison, schematic, and TEM imaging of sarcomere from patient without AF. AF; atrial fibrillation.

The findings of increased atrial fibrosis in zebrafish with TTNtv as well as compromised sarcomere structure associated with both *TTN* and *MYL4* variants, suggest a fundamental role of structural genes in AF and indicate a link between AF and cardiomyopathy. Characterizing genetic subtypes of cardiomyopathy and their associations with AF may help improve our understanding of the AF pathophysiology. Figure 3 illustrates how the AF cardiomyocyte is affected by fibrotic changes.

Our evolving knowledge of the genetic and structural basis of AF has led to new awareness that our current antiarrhythmic drugs might not target the major mechanisms implicated in AF. For many years, AF has been considered an electrical disease, but our understanding of the pathophysiology of AF has improved and it is now considered to be much more than an ion-channel disease. Atrial fibrosis has continuously been reported to be more frequent in patients with AF compared to non-AF patients [71].

In the absence of heart failure, patients with long-standing or persistent AF appear to have increased atrial fibrosis, whereas those with paroxysmal AF do not [72]. Cochet et al. found a higher degree of re-entrant activity in areas with atrial fibrosis in patients with persistent AF, and the authors propose this to be a likely AF mechanism [73].

In line with this, Mahnkopf et al. investigated atrial fibrosis as a marker for structural remodeling and found extensive structural remodeling in lone AF patients compared with non-lone AF patients [74]. In animals with long-standing AF, atrial fibrosis can be prevented by inhibition of the renin–angiotensin system, which appears to significantly reduce the duration of AF [75]. The potential use of angiotensin receptor blockers and angiotensin-converting-enzyme inhibitors could be an interesting option for preventing the promotion of AF by suppressing the development of structural remodeling. Boldt et al. previously reported that angiotensin-converting-enzyme inhibitors reduce fibrosis in patients with lone AF [76].

## Conclusion

Although extensive efforts have been made to identify the role of AF genetics in AF pathology, the field continues to grow as we explore new associations. During the last decades, variants in ion-channel genes e.g., in sodium and potassium-channel genes, and in non-ion-channel genes including structural genes have been associated with AF. Recently, both *TTN* and *MYL4* variants have been associated with early-onset AF. The discovery that early atrial fibrosis plays a significant role in atrial cardiomyopathy and in AF has given us a better understanding of the AF pathogenesis. Based on the differences in the pathogenesis of AF, the assumption of a “one-size-fits all” treatment is inadequate. AF as a polygenetic complex disease with a structural component is a new but very promising area of research and might prove very important in the future treatment of AF.

## Supplementary information

Supplementary material

Supplementary Table 1 - Common variants

Supplementary Table 2 - Rare variants
