# Supplementary material for: Atrial fibrillation—a complex polygenetic disease
Source: Eur J Hum Genet. 2020 Dec 5;29(7):1051–60. doi: 10.1038/s41431-020-00784-8 (PMC8298566; doi:10.1038/s41431-020-00784-8)
Supplement: Supplementary file 1 — Supplementary material [file 41431_2020_784_MOESM1_ESM.docx]

**SUPPLEMENTARY MATERIAL**

Atrial Fibrillation – A Complex Polygenetic Disease

Andersen and Andreasen et al.

**REFERENCES SUPPLEMENTARY TABLE 2**

1 Olson TM, Alekseev AE, Moreau C, Liu XK, Zingman LV, Miki T *et al.* KATP channel mutation confers risk for vein of Marshall adrenergic atrial fibrillation. *Nat Clin Pract Cardiovasc Med* 2007; **4**: 110–116.

2 Macri V, Mahida SN, Zhang ML, Sinner MF, Dolmatova EV, Tucker NR *et al.* A novel trafficking-defective HCN4 mutation is associated with early-onset atrial fibrillation. *Heart Rhythm* 2014; **11**: 1055–1062.

3 Christophersen IE, Olesen MS, Liang B, Andersen MN, Larsen AP, Nielsen JB *et al.* Genetic variation in KCNA5: impact on the atrial-specific potassium current IKur in patients with lone atrial fibrillation. *Eur Heart J* 2013; **34**: 1517–1525.

4 Yang T, Yang P, Roden DM, Darbar D. Novel KCNA5 mutation implicates tyrosine kinase signaling in human atrial fibrillation. *Heart Rhythm* 2010; **7**: 1246–1252.

5 Mann SA, Otway R, Guo G, Soka M, Karlsdotter L, Trivedi G *et al.* Epistatic effects of potassium channel variation on cardiac repolarization and atrial fibrillation risk. *J Am Coll Cardiol* 2012; **59**: 1017–1025.

6 Gregers E, Ahlberg G, Christensen T, Jabbari J, Larsen KO, Herfelt CB *et al.* Deep sequencing of atrial fibrillation patients with mitral valve regurgitation shows no evidence of mosaicism but reveals novel rare germline variants. *Heart Rhythm* 2017; **14**: 1531–1538.

7 Olson TM, Alekseev AE, Liu XK, Park S, Zingman LV, Bienengraeber M *et al.* Kv1.5 channelopathy due to KCNA5 loss-of-function mutation causes human atrial fibrillation. *Hum Mol Genet* 2006; **15**: 2185–2191.

8 Hayashi K, Konno T, Tada H, Tani S, Liu L, Fujino N *et al.* Functional Characterization of Rare Variants Implicated in Susceptibility to Lone Atrial Fibrillation. *Circ Arrhythm Electrophysiol* 2015; **8**: 1095–1104.

9 Yang Y, Li J, Lin X, Yang Y, Hong K, Wang L *et al.* Novel KCNA5 loss-of-function mutations responsible for atrial fibrillation. *J Hum Genet* 2009; **54**: 277–283.

10 Huang Y, Yang J, Xie W, Li Q, Zeng Z, Sui H *et al.* A novel KCND3 mutation associated with early-onset lone atrial fibrillation. *Oncotarget* 2017; **8**: 115503–115512.

11 Olesen MS, Refsgaard L, Holst AG, Larsen AP, Grubb S, Haunsø S *et al.* A novel KCND3 gain-of-function mutation associated with early-onset of persistent lone atrial fibrillation. *Cardiovasc Res* 2013; **98**: 488–495.

12 Olesen MS, Bentzen BH, Nielsen JB, Steffensen AB, David J-P, Jabbari J *et al.* Mutations in the potassium channel subunit KCNE1 are associated with early-onset familial atrial fibrillation. *BMC Med Genet* 2012; **13**: 24.

13 Yang Y, Xia M, Jin Q, Bendahhou S, Shi J, Chen Y *et al.* Identification of a KCNE2 gain-of-function mutation in patients with familial atrial fibrillation. *Am J Hum Genet* 2004; **75**: 899–905.

14 Nielsen JB, Bentzen BH, Olesen MS, David J-P, Olesen S-P, Haunsø S *et al.* Gain-of-function mutations in potassium channel subunit KCNE2 associated with early-onset lone atrial fibrillation. *Biomark Med* 2014; **8**: 557–570.

15 Lundby A, Ravn LS, Svendsen JH, Hauns S, Olesen S-P, Schmitt N. KCNE3 mutation V17M identified in a patient with lone atrial fibrillation. *Cell Physiol Biochem* 2008; **21**: 47–54.

16 Zhang D-F, Liang B, Lin J, Liu B, Zhou Q-S, Yang Y-Q. [KCNE3 R53H substitution in familial atrial fibrillation]. *Chin Med J* 2005; **118**: 1735–1738.

17 Ravn LS, Aizawa Y, Pollevick GD, Hofman-Bang J, Cordeiro JM, Dixen U *et al.* Gain of function in IKs secondary to a mutation in KCNE5 associated with atrial fibrillation. *Heart Rhythm* 2008; **5**: 427–435.

18 Hong K, Bjerregaard P, Gussak I, Brugada R. Short QT syndrome and atrial fibrillation caused by mutation in KCNH2. *J Cardiovasc Electrophysiol* 2005; **16**: 394–396.

19 Harrell DT, Ashihara T, Ishikawa T, Tominaga I, Mazzanti A, Takahashi K *et al.* Genotype-dependent differences in age of manifestation and arrhythmia complications in short QT syndrome. *Int J Cardiol* 2015; **190**: 393–402.

20 Doñate Puertas R, Millat G, Ernens I, Gache V, Chauveau S, Morel E *et al.* Atrial Structural Remodeling Gene Variants in Patients with Atrial Fibrillation. *Biomed Res Int* 2018; **2018**: 4862480.

21 Xia M, Jin Q, Bendahhou S, He Y, Larroque M-M, Chen Y *et al.* A Kir2.1 gain-of-function mutation underlies familial atrial fibrillation. *Biochem Biophys Res Commun* 2005; **332**: 1012–1019.

22 Deo M, Ruan Y, Pandit SV, Shah K, Berenfeld O, Blaufox A *et al.* KCNJ2 mutation in short QT syndrome 3 results in atrial fibrillation and ventricular proarrhythmia. *Proc Natl Acad Sci USA* 2013; **110**: 4291–4296.

23 Hattori T, Makiyama T, Akao M, Ehara E, Ohno S, Iguchi M *et al.* A novel gain-of-function KCNJ2 mutation associated with short-QT syndrome impairs inward rectification of Kir2.1 currents. *Cardiovasc Res* 2012; **93**: 666–673.

24 Calloe K, Ravn LS, Schmitt N, Sui JL, Duno M, Haunso S *et al.* Characterizations of a loss-of-function mutation in the Kir3.4 channel subunit. *Biochem Biophys Res Commun* 2007; **364**: 889–895.

25 Delaney JT, Muhammad R, Blair MA, Kor K, Fish FA, Roden DM *et al.* A KCNJ8 mutation associated with early repolarization and atrial fibrillation. *Europace* 2012; **14**: 1428–1432.

26 Liang B, Soka M, Christensen AH, Olesen MS, Larsen AP, Knop FK *et al.* Genetic variation in the two-pore domain potassium channel, TASK-1, may contribute to an atrial substrate for arrhythmogenesis. *J Mol Cell Cardiol* 2014; **67**: 69–76.

27 Tsai C-T, Hsieh C-S, Chang S-N, Chuang EY, Juang J-MJ, Lin L-Y *et al.* Next-generation sequencing of nine atrial fibrillation candidate genes identified novel de novo mutations in patients with extreme trait of atrial fibrillation. *J Med Genet* 2015; **52**: 28–36.

28 Otway R, Vandenberg JI, Guo G, Varghese A, Castro ML, Liu J *et al.* Stretch-sensitive KCNQ1 mutation A link between genetic and environmental factors in the pathogenesis of atrial fibrillation? *J Am Coll Cardiol* 2007; **49**: 578–586.

29 Abraham RL, Yang T, Blair M, Roden DM, Darbar D. Augmented potassium current is a shared phenotype for two genetic defects associated with familial atrial fibrillation. *J Mol Cell Cardiol* 2010; **48**: 181–190.

30 Chen Y-H, Xu S-J, Bendahhou S, Wang X-L, Wang Y, Xu W-Y *et al.* KCNQ1 gain-of-function mutation in familial atrial fibrillation. *Science* 2003; **299**: 251–254.

31 Righi D, Silvetti MS, Drago F. Sinus bradycardia, junctional rhythm, and low-rate atrial fibrillation in Short QT syndrome during 20 years of follow-up: three faces of the same genetic problem. *Cardiol Young* 2016; **26**: 589–592.

32 Das S, Makino S, Melman YF, Shea MA, Goyal SB, Rosenzweig A *et al.* Mutation in the S3 segment of KCNQ1 results in familial lone atrial fibrillation. *Heart Rhythm* 2009; **6**: 1146–1153.

33 Bartos DC, Anderson JB, Bastiaenen R, Johnson JN, Gollob MH, Tester DJ *et al.* A KCNQ1 mutation causes a high penetrance for familial atrial fibrillation. *J Cardiovasc Electrophysiol* 2013; **24**: 562–569.

34 Bartos DC, Duchatelet S, Burgess DE, Klug D, Denjoy I, Peat R *et al.* R231C mutation in KCNQ1 causes long QT syndrome type 1 and familial atrial fibrillation. *Heart Rhythm* 2011; **8**: 48–55.

35 Steffensen AB, Refsgaard L, Andersen MN, Vallet C, Mujezinovic A, Haunsø S *et al.* IKs Gain- and Loss-of-Function in Early-Onset Lone Atrial Fibrillation. *J Cardiovasc Electrophysiol* 2015; **26**: 715–723.

36 Lundby A, Ravn LS, Svendsen JH, Olesen S-P, Schmitt N. KCNQ1 mutation Q147R is associated with atrial fibrillation and prolonged QT interval. *Heart Rhythm* 2007; **4**: 1532–1541.

37 Hasegawa K, Ohno S, Ashihara T, Itoh H, Ding W-G, Toyoda F *et al.* A novel KCNQ1 missense mutation identified in a patient with juvenile-onset atrial fibrillation causes constitutively open IKs channels. *Heart Rhythm* 2014; **11**: 67–75.

38 Ki C-S, Jung CL, Kim H, Baek K-H, Park SJ, On YK *et al.* A KCNQ1 mutation causes age-dependant bradycardia and persistent atrial fibrillation. *Pflugers Arch* 2014; **466**: 529–540.

39 Darbar D, Kannankeril PJ, Donahue BS, Kucera G, Stubblefield T, Haines JL *et al.* Cardiac sodium channel (SCN5A) variants associated with atrial fibrillation. *Circulation* 2008; **117**: 1927–1935.

40 Olesen MS, Yuan L, Liang B, Holst AG, Nielsen N, Nielsen JB *et al.* High prevalence of long QT syndrome-associated SCN5A variants in patients with early-onset lone atrial fibrillation. *Circ Cardiovasc Genet* 2012; **5**: 450–459.

41 Olson TM, Michels VV, Ballew JD, Reyna SP, Karst ML, Herron KJ *et al.* Sodium channel mutations and susceptibility to heart failure and atrial fibrillation. *JAMA* 2005; **293**: 447–454.

42 Li Q, Huang H, Liu G, Lam K, Rutberg J, Green MS *et al.* Gain-of-function mutation of Nav1.5 in atrial fibrillation enhances cellular excitability and lowers the threshold for action potential firing. *Biochem Biophys Res Commun* 2009; **380**: 132–137.

43 Makiyama T, Akao M, Shizuta S, Doi T, Nishiyama K, Oka Y *et al.* A novel SCN5A gain-of-function mutation M1875T associated with familial atrial fibrillation. *J Am Coll Cardiol* 2008; **52**: 1326–1334.

44 Ellinor PT, Nam EG, Shea MA, Milan DJ, Ruskin JN, MacRae CA. Cardiac sodium channel mutation in atrial fibrillation. *Heart Rhythm* 2008; **5**: 99–105.

45 Husser D, Ueberham L, Hindricks G, Büttner P, Ingram C, Weeke P *et al.* Rare variants in genes encoding the cardiac sodium channel and associated compounds and their impact on outcome of catheter ablation of atrial fibrillation. *PLoS ONE* 2017; **12**: e0183690.

46 Chen LY, Ballew JD, Herron KJ, Rodeheffer RJ, Olson TM. A common polymorphism in SCN5A is associated with lone atrial fibrillation. *Clin Pharmacol Ther* 2007; **81**: 35–41.

47 Boddum K, Saljic A, Jespersen T, Christensen AH. A Novel SCN5A Variant Associated with Abnormal Repolarization, Atrial Fibrillation, and Reversible Cardiomyopathy. *Cardiology* 2018; **140**: 8–13.

48 Musa H, Kline CF, Sturm AC, Murphy N, Adelman S, Wang C *et al.* SCN5A variant that blocks fibroblast growth factor homologous factor regulation causes human arrhythmia. *Proc Natl Acad Sci USA* 2015; **112**: 12528–12533.

49 Lieve KV, Verkerk AO, Podliesna S, van der Werf C, Tanck MW, Hofman N *et al.* Gain-of-function mutation in SCN5A causes ventricular arrhythmias and early onset atrial fibrillation. *Int J Cardiol* 2017; **236**: 187–193.

50 Ziyadeh-Isleem A, Clatot J, Duchatelet S, Gandjbakhch E, Denjoy I, Hidden-Lucet F *et al.* A truncating SCN5A mutation combined with genetic variability causes sick sinus syndrome and early atrial fibrillation. *Heart Rhythm* 2014; **11**: 1015–1023.

51 Yagihara N, Watanabe H, Barnett P, Duboscq-Bidot L, Thomas AC, Yang P *et al.* Variants in the SCN5A Promoter Associated With Various Arrhythmia Phenotypes. *J Am Heart Assoc* 2016; **5**: e003644.

52 Savio-Galimberti E, Weeke P, Muhammad R, Blair M, Ansari S, Short L *et al.* SCN10A/Nav1.8 modulation of peak and late sodium currents in patients with early onset atrial fibrillation. *Cardiovasc Res* 2014; **104**: 355–363.

53 Jabbari J, Olesen MS, Yuan L, Nielsen JB, Liang B, Macri V *et al.* Common and rare variants in SCN10A modulate the risk of atrial fibrillation. *Circ Cardiovasc Genet* 2015; **8**: 64–73.

54 Watanabe H, Darbar D, Kaiser DW, Jiramongkolchai K, Chopra S, Donahue BS *et al.* Mutations in sodium channel β1- and β2-subunits associated with atrial fibrillation. *Circ Arrhythm Electrophysiol* 2009; **2**: 268–275.

55 Olesen MS, Holst AG, Svendsen JH, Haunsø S, Tfelt-Hansen J. SCN1Bb R214Q found in 3 patients: 1 with Brugada syndrome and 2 with lone atrial fibrillation. *Heart Rhythm* 2012; **9**: 770–773.

56 Hu D, Barajas-Martínez H, Medeiros-Domingo A, Crotti L, Veltmann C, Schimpf R *et al.* A novel rare variant in SCN1Bb linked to Brugada syndrome and SIDS by combined modulation of Na(v)1.5 and K(v)4.3 channel currents. *Heart Rhythm* 2012; **9**: 760–769.

57 Olesen MS, Jespersen T, Nielsen JB, Liang B, Møller DV, Hedley P *et al.* Mutations in sodium channel β-subunit SCN3B are associated with early-onset lone atrial fibrillation. *Cardiovasc Res* 2011; **89**: 786–793.

58 Wang P, Yang Q, Wu X, Yang Y, Shi L, Wang C *et al.* Functional dominant-negative mutation of sodium channel subunit gene SCN3B associated with atrial fibrillation in a Chinese GeneID population. *Biochem Biophys Res Commun* 2010; **398**: 98–104.

59 Li R-G, Wang Q, Xu Y-J, Zhang M, Qu X-K, Liu X *et al.* Mutations of the SCN4B-encoded sodium channel β4 subunit in familial atrial fibrillation. *Int J Mol Med* 2013; **32**: 144–150.

60 Girolami F, Iascone M, Tomberli B, Bardi S, Benelli M, Marseglia G *et al.* Novel α-actinin 2 variant associated with familial hypertrophic cardiomyopathy and juvenile atrial arrhythmias: a massively parallel sequencing study. *Circ Cardiovasc Genet* 2014; **7**: 741–750.

61 Tsai C-T, Chang S-N, Chang S-H, Lee J-K, Lin L-Y, Wu C-K *et al.* Renin-angiotensin system gene polymorphisms predict the risk of stroke in patients with atrial fibrillation: a 10-year prospective follow-up study. *Heart Rhythm* 2014; **11**: 1384–1390.

62 Seppälä I, Kleber ME, Bevan S, Lyytikäinen L-P, Oksala N, Hernesniemi JA *et al.* Associations of functional alanine-glyoxylate aminotransferase 2 gene variants with atrial fibrillation and ischemic stroke. *Sci Rep* 2016; **6**: 23207.

63 Jiang J-Q, Shen F-F, Fang W-Y, Liu X, Yang Y-Q. Novel GATA4 mutations in lone atrial fibrillation. *Int J Mol Med* 2011; **28**: 1025–1032.

64 Wang J, Sun Y-M, Yang Y-Q. Mutation spectrum of the GATA4 gene in patients with idiopathic atrial fibrillation. *Mol Biol Rep* 2012; **39**: 8127–8135.

65 Yang Y-Q, Wang M-Y, Zhang X-L, Tan H-W, Shi H-F, Jiang W-F *et al.* GATA4 loss-of-function mutations in familial atrial fibrillation. *Clin Chim Acta* 2011; **412**: 1825–1830.

66 Posch MG, Boldt L-H, Polotzki M, Richter S, Rolf S, Perrot A *et al.* Mutations in the cardiac transcription factor GATA4 in patients with lone atrial fibrillation. *Eur J Med Genet* 2010; **53**: 201–203.

67 Gu J-Y, Xu J-H, Yu H, Yang Y-Q. Novel GATA5 loss-of-function mutations underlie familial atrial fibrillation. *Clinics (Sao Paulo)* 2012; **67**: 1393–1399.

68 Yang Y-Q, Wang J, Wang X-H, Wang Q, Tan H-W, Zhang M *et al.* Mutational spectrum of the GATA5 gene associated with familial atrial fibrillation. *Int J Cardiol* 2012; **157**: 305–307.

69 Mahida S. Transcription factors and atrial fibrillation. *Cardiovasc Res* 2014; **101**: 194–202.

70 Wang X-H, Huang C-X, Wang Q, Li R-G, Xu Y-J, Liu X *et al.* A novel GATA5 loss-of-function mutation underlies lone atrial fibrillation. *Int J Mol Med* 2013; **31**: 43–50.

71 Yang Y-Q, Wang X-H, Tan H-W, Jiang W-F, Fang W-Y, Liu X. Prevalence and spectrum of GATA6 mutations associated with familial atrial fibrillation. *Int J Cardiol* 2012; **155**: 494–496.

72 Tucker NR, Mahida S, Ye J, Abraham EJ, Mina JA, Parsons VA *et al.* Gain-of-function mutations in GATA6 lead to atrial fibrillation. *Heart Rhythm* 2017; **14**: 284–291.

73 Yang Y-Q, Li L, Wang J, Zhang X-L, Li R-G, Xu Y-J *et al.* GATA6 loss-of-function mutation in atrial fibrillation. *Eur J Med Genet* 2012; **55**: 520–526.

74 Li J, Liu W-D, Yang Z-L, Yang Y-Q. Novel GATA6 loss-of-function mutation responsible for familial atrial fibrillation. *Int J Mol Med* 2012; **30**: 783–790.

75 Thibodeau IL, Xu J, Li Q, Liu G, Lam K, Veinot JP *et al.* Paradigm of genetic mosaicism and lone atrial fibrillation: physiological characterization of a connexin 43-deletion mutant identified from atrial tissue. *Circulation* 2010; **122**: 236–244.

76 Yang Y-Q, Zhang X-L, Wang X-H, Tan H-W, Shi H-F, Jiang W-F *et al.* Connexin40 nonsense mutation in familial atrial fibrillation. *Int J Mol Med* 2010; **26**: 605–610.

77 Sun Y, Yang Y-Q, Gong X-Q, Wang X-H, Li R-G, Tan H-W *et al.* Novel germline GJA5/connexin40 mutations associated with lone atrial fibrillation impair gap junctional intercellular communication. *Hum Mutat* 2013; **34**: 603–609.

78 Yang Y-Q, Liu X, Zhang X-L, Wang X-H, Tan H-W, Shi H-F *et al.* Novel connexin40 missense mutations in patients with familial atrial fibrillation. *Europace* 2010; **12**: 1421–1427.

79 Gollob MH, Jones DL, Krahn AD, Danis L, Gong X-Q, Shao Q *et al.* Somatic mutations in the connexin 40 gene (GJA5) in atrial fibrillation. *N Engl J Med* 2006; **354**: 2677–2688.

80 Shi H-F, Yang J-F, Wang Q, Li R-G, Xu Y-J, Qu X-K *et al.* Prevalence and spectrum of GJA5 mutations associated with lone atrial fibrillation. *Mol Med Rep* 2013; **7**: 767–774.

81 Müller II, Melville DB, Tanwar V, Rybski WM, Mukherjee A, Shoemaker MB *et al.* Functional modeling in zebrafish demonstrates that the atrial-fibrillation-associated gene GREM2 regulates cardiac laterality, cardiomyocyte differentiation and atrial rhythm. *Dis Model Mech* 2013; **6**: 332–341.

82 Beavers DL, Wang W, Ather S, Voigt N, Garbino A, Dixit SS *et al.* Mutation E169K in junctophilin-2 causes atrial fibrillation due to impaired RyR2 stabilization. *J Am Coll Cardiol* 2013; **62**: 2010–2019.

83 Beckmann BM, Holinski-Feder E, Walter MC, Haserück N, Reithmann C, Hinterseer M *et al.* Laminopathy presenting as familial atrial fibrillation. *Int J Cardiol* 2010; **145**: 394–396.

84 Brauch KM, Chen LY, Olson TM. Comprehensive mutation scanning of LMNA in 268 patients with lone atrial fibrillation. *Am J Cardiol* 2009; **103**: 1426–1428.

85 Saj M, Dabrowski R, Labib S, Jankowska A, Szperl M, Broda G *et al.* Variants of the lamin A/C (LMNA) gene in non-valvular atrial fibrillation patients: a possible pathogenic role of the Thr528Met mutation. *Mol Diagn Ther* 2012; **16**: 99–107.

86 Hoorntje ET, Bollen IA, Barge-Schaapveld DQ, van Tienen FH, Te Meerman GJ, Jansweijer JA *et al.* Lamin A/C-Related Cardiac Disease: Late Onset With a Variable and Mild Phenotype in a Large Cohort of Patients With the Lamin A/C p.(Arg331Gln) Founder Mutation. *Circ Cardiovasc Genet* 2017; **10**: e001631.

87 Zhao J, Yao H, Li Z, Wang L, Liu G, Wang DW *et al.* A novel nonsense mutation in LMNA gene identified by Exome Sequencing in an atrial fibrillation family. *Eur J Med Genet* 2016; **59**: 396–400.

88 Thorolfsdottir RB, Sveinbjornsson G, Sulem P, Nielsen JB, Jonsson S, Halldorsson GH *et al.* Coding variants in RPL3L and MYZAP increase risk of atrial fibrillation. *Commun Biol* 2018; **1**: 68.

89 Gudbjartsson DF, Holm H, Sulem P, Masson G, Oddsson A, Magnusson OT *et al.* A frameshift deletion in the sarcomere gene MYL4 causes early-onset familial atrial fibrillation. *Eur Heart J* 2017; **38**: 27–34.

90 Orr N, Arnaout R, Gula LJ, Spears DA, Leong-Sit P, Li Q *et al.* A mutation in the atrial-specific myosin light chain gene (MYL4) causes familial atrial fibrillation. *Nat Commun* 2016; **7**: 11303.

91 Lee JS, Ko Y-G, Shin K-J, Kim S-K, Park JH, Hwang K-C *et al.* Mitochondrial DNA 4977bp deletion mutation in peripheral blood reflects atrial remodeling in patients with non-valvular atrial fibrillation. *Yonsei Med J* 2015; **56**: 53–61.

92 Ritchie MD, Rowan S, Kucera G, Stubblefield T, Blair M, Carter S *et al.* Chromosome 4q25 variants are genetic modifiers of rare ion channel mutations associated with familial atrial fibrillation. *J Am Coll Cardiol* 2012; **60**: 1173–1181.

93 Xie W-H, Chang C, Xu Y-J, Li R-G, Qu X-K, Fang W-Y *et al.* Prevalence and spectrum of Nkx2.5 mutations associated with idiopathic atrial fibrillation. *Clinics (Sao Paulo)* 2013; **68**: 777–784.

94 Yu H, Xu J-H, Song H-M, Zhao L, Xu W-J, Wang J *et al.* Mutational spectrum of the NKX2-5 gene in patients with lone atrial fibrillation. *Int J Med Sci* 2014; **11**: 554–563.

95 Yuan F, Qiu X-B, Li R-G, Qu X-K, Wang J, Xu Y-J *et al.* A novel NKX2-5 loss-of-function mutation predisposes to familial dilated cardiomyopathy and arrhythmias. *Int J Mol Med* 2015; **35**: 478–486.

96 Wang J, Zhang D-F, Sun Y-M, Li R-G, Qiu X-B, Qu X-K *et al.* NKX2-6 mutation predisposes to familial atrial fibrillation. *Int J Mol Med* 2014; **34**: 1581–1590.

97 Fatini C, Sticchi E, Genuardi M, Sofi F, Gensini F, Gori AM *et al.* Analysis of minK and eNOS genes as candidate loci for predisposition to non-valvular atrial fibrillation. *Eur Heart J* 2006; **27**: 1712–1718.

98 Hodgson-Zingman DM, Karst ML, Zingman LV, Heublein DM, Darbar D, Herron KJ *et al.* Atrial natriuretic peptide frameshift mutation in familial atrial fibrillation. *N Engl J Med* 2008; **359**: 158–165.

99 Ren X, Xu C, Zhan C, Yang Y, Shi L, Wang F *et al.* Identification of NPPA variants associated with atrial fibrillation in a Chinese GeneID population. *Clin Chim Acta* 2010; **411**: 481–485.

100 Zhang X, Chen S, Yoo S, Chakrabarti S, Zhang T, Ke T *et al.* Mutation in nuclear pore component NUP155 leads to atrial fibrillation and early sudden cardiac death. *Cell* 2008; **135**: 1017–1027.

101 Qiu X-B, Xu Y-J, Li R-G, Xu L, Liu X, Fang W-Y *et al.* PITX2C loss-of-function mutations responsible for idiopathic atrial fibrillation. *Clinics (Sao Paulo)* 2014; **69**: 15–22.

102 Wang J, Zhang D-F, Sun Y-M, Yang Y-Q. A novel PITX2c loss-of-function mutation associated with familial atrial fibrillation. *Eur J Med Genet* 2014; **57**: 25–31.

103 Sternick EB, Oliva A, Gerken LM, Magalhães L, Scarpelli R, Correia FS *et al.* Clinical, electrocardiographic, and electrophysiologic characteristics of patients with a fasciculoventricular pathway: the role of PRKAG2 mutation. *Heart Rhythm* 2011; **8**: 58–64.

104 Bhuiyan ZA, van den Berg MP, van Tintelen JP, Bink-Boelkens MTE, Wiesfeld ACP, Alders M *et al.* Expanding spectrum of human RYR2-related disease: new electrocardiographic, structural, and genetic features. *Circulation* 2007; **116**: 1569–1576.

105 Kazemian P, Gollob MH, Pantano A, Oudit GY. A novel mutation in the RYR2 gene leading to catecholaminergic polymorphic ventricular tachycardia and paroxysmal atrial fibrillation: dose-dependent arrhythmia-event suppression by β-blocker therapy. *Can J Cardiol* 2011; **27**: 870.e7–10.

106 Di Pino A, Caruso E, Costanzo L, Guccione P. A novel RyR2 mutation in a 2-year-old baby presenting with atrial fibrillation, atrial flutter, and atrial ectopic tachycardia. *Heart Rhythm* 2014; **11**: 1480–1483.

107 Hoffmann S, Clauss S, Berger IM, Weiß B, Montalbano A, Röth R *et al.* Coding and non-coding variants in the SHOX2 gene in patients with early-onset atrial fibrillation. *Basic Res Cardiol* 2016; **111**: 36.

108 Nyberg MT, Stoevring B, Behr ER, Ravn LS, McKenna WJ, Christiansen M. The variation of the sarcolipin gene (SLN) in atrial fibrillation, long QT syndrome and sudden arrhythmic death syndrome. *Clin Chim Acta* 2007; **375**: 87–91.

109 Ma J-F, Yang F, Mahida SN, Zhao L, Chen X, Zhang ML *et al.* TBX5 mutations contribute to early-onset atrial fibrillation in Chinese and Caucasians. *Cardiovasc Res* 2016; **109**: 442–450.

110 Postma AV, van de Meerakker JBA, Mathijssen IB, Barnett P, Christoffels VM, Ilgun A *et al.* A gain-of-function TBX5 mutation is associated with atypical Holt-Oram syndrome and paroxysmal atrial fibrillation. *Circ Res* 2008; **102**: 1433–1442.

111 Guo D-F, Li R-G, Yuan F, Shi H-Y, Hou X-M, Qu X-K *et al.* TBX5 loss-of-function mutation contributes to atrial fibrillation and atypical Holt-Oram syndrome. *Mol Med Rep* 2016; **13**: 4349–4356.

112 Wang Z-C, Ji W-H, Ruan C-W, Liu X-Y, Qiu X-B, Yuan F *et al.* Prevalence and Spectrum of TBX5 Mutation in Patients with Lone Atrial Fibrillation. *Int J Med Sci* 2016; **13**: 60–67.

113 Theis JL, Zimmermann MT, Larsen BT, Rybakova IN, Long PA, Evans JM *et al.* TNNI3K mutation in familial syndrome of conduction system disease, atrial tachyarrhythmia and dilated cardiomyopathy. *Hum Mol Genet* 2014; **23**: 5793–5804.

114 Wang C, Wu M, Qian J, Li B, Tu X, Xu C *et al.* Identification of rare variants in TNNI3 with atrial fibrillation in a Chinese GeneID population. *Mol Genet Genomics* 2016; **291**: 79–92.

115 Oberti C, Wang L, Li L, Dong J, Rao S, Du W *et al.* Genome-wide linkage scan identifies a novel genetic locus on chromosome 5p13 for neonatal atrial fibrillation associated with sudden death and variable cardiomyopathy. *Circulation* 2004; **110**: 3753–3759.

116 Vad OB, Paludan-Müller C, Ahlberg G, Kalstø SM, Ghouse J, Andreasen L *et al.* Loss-of-Function Variants in Cytoskeletal Genes Are Associated with Early-Onset Atrial Fibrillation. *Journal of Clinical Medicine* 2020; **9**: 372.
